# Supplementary material for: Catalases in the pathogenesis of Sporothrix schenckii research
Source: PeerJ. 2022 Dec 7;10:e14478. doi: 10.7717/peerj.14478 (PMC9745942; doi:10.7717/peerj.14478)
Supplement: Supplemental Information 1 [file peerj-10-14478-s001.docx]

| **Supplementary Table 1**. Representative articles cited in this manuscript, listed alphabetically. | | |
| --- | --- | --- |
| *Main author* | *Summary* | *Reference(s)* |
| Aguirre, J. | In this review, Aguirre et al., provides a summary of the current knowledge of fungal responses to ROS. This review focuses also in the human pathogen *Aspergillus fumigatus*, and the role of the antioxidant mechanisms in pathogenesis. This review serves as a guide for the proteins involved in the antioxidant mechanisms in fungi. | Aguirre, J., Hansberg, W., Navarro, R. (2006). Fungal responses to reactive oxygen species. Med Mycol. 1;44(Supplement_1):S101-S107. doi: 10.1080/13693780600900080 |
| Aguirre, J. | In this review, authors provide a review of the roles of ROS in cell differentiation and its importance in physiological roles in fungi. | Aguirre, J., Ríos-Momberg, M., Hewitt, D., Hansberg, W. (2005). Reactive oxygen species and development in microbial eukaryotes. Trends Microbiol. 13(3):111-8. doi: 10.1016/j.tim.2005.01.007. |
| Almeida, M.A. | Here, authors demonstrated that catalase P can be used as a marker for the diagnosis of *H. capsulatum* infection. Also, they provide information regarding the possible role of catalase and other proteins in fungal pathogenesis. | Almeida, M.A., Almeida-Paes, R., Guimarães, A.J., Valente, R.H., Soares, C.M.A., Zancopé-Oliveira. R.M. (2020). Immunoproteomics reveals pathogen's antigens involved in *Homo sapiens*-*Histoplasma capsulatum* interaction and specific linear B-cell epitopes in histoplasmosis. Front Cell Infect Microbiol. 29;10:591121. doi: 10.3389/fcimb.2020.591121. |
| Asemoloye, M.D. | Authors demonstrate that environmentally isolated fungal strains from contaminated soil with crude oil, showed increased transcriptomic and enzymatic activities related to oxidative stress and cellulose metabolism. This work sets the foundation of the use of strains with these features that may be useful for bioremediation of contaminated sites. | Asemoloye, M.D., Ahmad, R., Jonathan, S.G. (2018). Transcriptomic responses of catalase, peroxidase and laccase encoding genes and enzymatic activities of oil spill inhabiting rhizospheric fungal strains. Environ Pollut. 235:55-64. doi: 10.1016/j.envpol.2017.12.042. |
| Brancini, G-T.P. | Authors provide a view of photobiology and the response to stress in a entomopathogenic fungus. | Brancini, G.T.P., Hallsworth, J.E., Corrochano, L.M., Braga, G.Ú.L. (2022). Photobiology of the keystone genus *Metarhizium*. J Photochem Photobiol B. 226:112374. doi: 10.1016/j.jphotobiol.2021. |
| Caceres, I. | Here, piperine is shown to downregulate the production of aflatoxin by *A. flavus*, but also, they show that other biosynthetic pathways are negatively regulated, among those, the antioxidant enzymes catalase and superoxide dismutase. | Caceres, I., El Khoury, R., Bailly, S., Oswald, I.P., Puel, O., Bailly, J.D. (2017) Piperine inhibits aflatoxin B1 production in *Aspergillus flavus* by modulating fungal oxidative stress response. Fungal Genet Biol. 107:77-85. doi: 10.1016/j.fgb.2017.08.005. |
| Castaño, J. | Relevance of ROS for wood degradation and how ROS and the resistance mechanisms have been neglected of our current knowledge of wood decay and rot. | Castaño, J., Zhang, J., Zhou, M., Tsai, C.F., Lee, J.Y., Nicora, C., Schilling, J. (2021). A Fungal Secretome Adapted for Stress Enabled a Radical Wood Decay Mechanism. mBio. 31;12(4):e0204021. doi: 10.1128/mBio.02040-21.  Castaño, J.D., Zhang, J., Anderson, C.E,, Schilling, J.S. (2018). Oxidative Damage Control during Decay of Wood by Brown Rot Fungus Using Oxygen Radicals. Appl Environ Microbiol. 30;84(22):e01937-18. doi: 10.1128/AEM.01937-18. |
| Castro, V.S.P. | Describes the overall effect of *S. schenckii* infection in rats, highlighting the imbalance in redox state of the host and the increase of antioxidant enzymes. This study is relevant for the pathogenesis of this organism. | Castro, V.S.P., Da Silva, A.S., Thomé, G.R., Wolkmer, P., Castro, J.L.C., Costa, M.M., Graça, D.L., Oliveira, D.C., Alves, S.H., Schetinger, M.R.C., Lopes, S.T.A., Stefani, L.M., Azevedo, M.I., Baldissera, M.D., Andrade, C.M. (2017). Oxidative stress in rats experimentally infected by *Sporothrix schenckii*. Microb Pathog. 2017 Jun;107:1-5. doi: 10.1016/j.micpath.2017.03.001. |
| Dai, J. | Proposes the interaction of metformin with catalase and may be involved in reducing oxidative liver damage. | Dai, J., Liu, M., Ai, Q., Lin, L., Wu, K., Deng, X., Jing, Y., Jia, M., Wan, J., Zhang, L. (2014). Involvement of catalase in the protective benefits of metformin in mice with oxidative liver injury. Chem Biol Interact. 5;216:34-42. doi: 10.1016/j.cbi.2014.03.013. |
| De Groot, H. | Shows relevant mechanistic differences in bovine liver catalase by not releasing oxygen in the dismutation of hydrogen peroxide. | de Groot, H., Auferkamp, O., Bramey, T., de Groot, K., Kirsch, M., Korth, H.G., Petrat, F., Sustmann, R. (2006). Non-oxygen-forming pathways of hydrogen peroxide degradation by bovine liver catalase at low hydrogen peroxide fluxes. Free Radic Res. 2006;40:67–74. |
| De Souza, P.C. | Highlights the importance of alternative infection models for pathogenic fungi. Here, the invertebrate *Tenebrio molitor* is shown to be a good host model to measure the effects of fungal infection by *Candida albicans* or *Cryptococcus neoformans*. These models can be used to assess preliminary the effect of mutations in the infection process. | de Souza, P.C., Morey, A.T., Castanheira, G.M., Bocate, K.P., Panagio, L.A., Ito, F.A., Furlaneto, M.C., Yamada-Ogatta, S.F., Costa, I.N., Mora-Montes, H.M., Almeida, R.S. (2015). *Tenebrio molitor* (Coleoptera: Tenebrionidae) as an alternative host to study fungal infections. J Microbiol Methods. 118:182-6. doi: 10.1016/j.mimet.2015.10.004. |
| Díaz, A. | Two very important structural studies of *N. crassa* large-subunit catalase, showing mechanistic features, active site properties and the review of the structure-function relationships. | Díaz, A., Muñoz-Clares, R.A., Rangel, P., Valdés, V.J., Hansberg, W. (2005). Functional and structural analysis of catalase oxidized by singlet oxygen. Biochimie. 87(2):205-14. doi: 10.1016/j.biochi.2004.10.014.  Díaz, A., Valdés, V.J., Rudiño-Piñera, E., Horjales, E., Hansberg, W. (2009). Structure-function relationships in fungal large-subunit catalases. J Mol Biol. 13;386(1):218-32. doi: 10.1016/j.jmb.2008.12.019. |
| Domínguez, L. | Structural studies show the relevant residues needed to exclude water from the active site, concentrate, and provide the specificity for hydrogen peroxide. | Domínguez, L., Sosa-Peinado, A., Hansberg, W. (2010). Catalase evolved to concentrate H_2_O_2_ at its active site. Arch Biochem Biophys. 1;500(1):82-91. doi: 10.1016/j.abb.2010.05.017  Domínguez, L., Sosa-Peinado, A., Hansberg, W. (2014). How catalase recognizes H₂O₂ in a sea of water. Proteins. 82(1):45-56. doi: 10.1002/prot.24352. |
| Egan, M.J. | Study addressing the role of reactive oxygen species produced *by Magnaporthe grisea* and its role in the pathogenic process in rice. The most relevant finding is the induction of appressoria formation by ROS production. | Egan, M.J., Wang, Z.Y., Jones, M.A., Smirnoff, N., Talbot, N.J. (2007). Generation of reactive oxygen species by fungal NADPH oxidases is required for rice blast disease. Proc Natl Acad Sci U S A. 10;104(28):11772-7. doi: 10.1073/pnas.0700574104. |
| Félix-Contreras, C. | Proteomic study that showed the presence of metabolic enzymes such as threalase, chitinase, glycoside hydrolase induced by oxidative stress. This study shows that other enzymes may be involved in ROS resistance in *S. schenckii*. | Félix-Contreras, C., Alba-Fierro, C.A., Ríos-Castro, E., Luna-Martínez, F., Cuéllar-Cruz, M., Ruiz-Baca, E. (2020). Proteomic analysis of *Sporothrix schenckii* cell wall reveals proteins involved in oxidative stress response induced by menadione. Microb Pathog. 141:103987. doi: 10.1016/j.micpath.2020.103987. |
| Giosa, D. | Transcriptomic analysis of *S. schenckii* mycelial and yeast forms. Also, the foundation of the *Sporothrix* genome database to access transcriptomic and genomic data. | Giosa, D., Felice, M.R., Giuffrè, L., Aiese Cigliano, R., Paytuví-Gallart, A., Lo Passo, C., Barresi, C., D'Alessandro, E., Huang, H., Criseo, G., Mora-Montes, H.M., de Hoog, S., Romeo, O. (2020). Transcriptome-wide expression profiling of *Sporothrix schenckii* yeast and mycelial forms and the establishment of the *Sporothrix* Genome DataBase. Microb Genom. 6(10):mgen000445. doi: 10.1099/mgen.0.000445. |
| Gremião, I.D.F. | Guidelines for the prevention and control of *S. brasiliensis*. Importance of zoonosis is highlighted. | Gremião, I.D.F., Martins da Silva da Rocha, E., Montenegro, H., Carneiro, A.J.B., Xavier, M.O., de Farias, M.R., Monti, F., Mansho, W., de Macedo Assunção Pereira, R.H., Pereira, S.A., Lopes-Becerra, L.M. (2021) Guideline for the management of feline sporotrichosis caused by *Sporothrix brasiliensis* and literature revision. Braz. J. Microbiol. 52:107–124. doi: 10.1007/s42770-020-00365-3. |
| Hansberg, W. | This report addresses the role of the C-terminal domain of the large subunit catalase having chaperon activity. This report is seminal for the finding that this domain can counteract protein misfolding caused by oxidative stress. | Hansberg, W., Nava-Ramírez, T., Rangel-Silva, P., Díaz-Vilchis, A., Mendoza-Oliva, A. (2022) Large-Size Subunit Catalases Are Chimeric Proteins: A H_2_O_2_ Selecting Domain with Catalase Activity Fused to a Hsp31-Derived Domain Conferring Protein Stability and Chaperone Activity. Antioxidants (Basel). 17;11(5):979. doi: 10.3390/antiox11050979. |
| Hernández-Oñate, M.A. | This report shows the upregulation of catalases in *Trichoderma atroviride* upon cell physical damage, as part of the mechanism counteracting cell injury. | Hernández-Oñate, M.A., Esquivel-Naranjo, E.U., Mendoza-Mendoza, A., Stewart, A., Herrera-Estrella, A.H. (2012). An injury-response mechanism conserved across kingdoms determines entry of the fungus *Trichoderma atroviride* into development. Proc Natl Acad Sci U S A. 11;109(37):14918-23. doi: 10.1073/pnas.1209396109. |
| Holbrook, E. D. | This study shows that two catalases that eliminate extra and intracellular ROS, are needed to efficiently survive immune system derived ROS. Here, both enzymes seem to be redundant. | Holbrook, E. D., Smolnycki, K. A., Youseff, B. H., and Rappleye, C. A. (2013). Redundant catalases detoxify phagocyte reactive oxygen and facilitate *Histoplasma capsulatum* pathogenesis. Infect. Immun. 81, 2334–2346. doi: 10.1128/IAI.00173-13 |
| Huang, Z. | This study shows that the deletion of the catalase gene in *Sclerotinia sclerotiorum*, shows sensitivity to hyperosmotic stress and membrane perturbing agents as well as increased sensitivity to fungicide Qol, suggesting a broader role of catalases. | Huang, Z., Lu, J., Liu, R., Wang, P., Hu, Y., Fang, A., Yang, Y., Qing, L., Bi, C., Yu, Y. (2021). SsCat2 encodes a catalase that is critical for the antioxidant response, QoI fungicide sensitivity, and pathogenicity of *Sclerotinia sclerotiorum*. Fungal Genet Biol. 149:103530. doi: 10.1016/j.fgb.2021.103530. |
| Johnson, C. H. | Here, authors shown that the two catalases in *Histoplasma capsulatum* are expressed in all cell morphologies and during oxidative stress but not by carbon source. | Johnson, C. H., Klotz, M. G., York, J. L., Kruft, V., and McEwen, J. E. (2002). Redundancy, phylogeny and differential expression of *Histoplasma capsulatum* catalases. Microbiology 148, 1129–1142. doi: 10.1099/00221287-148-4-1129. |
| Kamlárová, A. | This report discusses the evolution of a fungal catalase with bacterial origin in the thermoresistant fungus *Chaetomium thermophilum.* | Kamlárová, A., Chovanová, K., Zámocký, M. (2018). Peculiar genes for thermostable bifunctional catalase-peroxidases in *Chaetomium thermophilum* and their molecular evolution. Gene. 5;666:83-91. doi: 10.1016/j.gene.2018.05.007. |
| Karakus, Y.Y. | Seminal review on catalases from the functional and structural point of view. | Karakus, Y. Y. (2020). Typical Catalases: Function and Structure. In: Bagatini, M. D., editor. Glutathione System and Oxidative Stress in Health and Disease. London: IntechOpen. |
| Kim, S.Y. | In this study, authors demonstrate the deleterious effect on SOD and catalases when exposed to methylene blue or rose Bengal followed by light activation. This may provide alternatives for superficial fungal control. | Kim, S.Y., Kwon, O.J., Park, J.W. (2001). Inactivation of catalase and superoxide dismutase by singlet oxygen derived from photoactivated dye. Biochimie. 83(5):437-44. doi: 10.1016/s0300-9084(01)01258-5. |
| Krych, J. | Authors show inhibition of catalase by flavonoids, suggesting additional resources for fungi control. | Krych, J., Gebicka, L. (2013). Catalase is inhibited by flavonoids. Int J Biol Macromol. 58:148-53. doi: 10.1016/j.ijbiomac.2013.03.070. |
| Kwok, L.Y. | Authors showed that in the parasite *Toxoplasma gondii*, only one catalase is present and contributes to virulence. | Kwok, L.Y., Schlüter, D., Clayton, C., Soldati, D. (2004). The antioxidant systems in *Toxoplasma gondii* and the role of cytosolic catalase in defence against oxidative injury. Mol Microbiol. 51:47–61. doi.org/10.1046/j.1365-2958.2003.03823.x. |
| Li, G. | Here, authors demonstrate also that appressoria formation is linked to catalase expression in *Metharizium acridum* and, is needed for virulence. The cuticle and host defense systems posse a threat to this organism, therefore, the catalase plays a central role. | Li, G., Fan, A., Peng, G., Keyhani, N.O., Xin, J., Cao, Y., Xia, Y. (2017). A bifunctional catalase-peroxidase, MakatG1, contributes to the virulence of *Metarhizium acridum* by overcoming oxidative stress on the host insect cuticle. Environ Microbiol. 19(10):4365-4378. doi: 10.1111/1462-2920.13932. |
| Ma, X. | A seminal review on the activators and inhibitors of antioxidant enzymes. | Ma, X., Deng, D., Chen, W. (2017). Inhibitors and Activators of SOD, GSH‐Px, and CAT. In: Senturk, M. , editor. Enzyme Inhibitors and Activators [Internet]. London: IntechOpen; doi: 10.5772/65936. |
| Michán, S. | The demonstration that ROS and development are tightly linked in *Neurospora crassa*. | Michán, S., Lledías, F., Hansberg, W. (2003). Asexual development is increased in *Neurospora crassa cat-3*-null mutant strains. Eukaryot Cell. 2(4):798-808. doi: 10.1128/EC.2.4.798-808.2003. |
| Nava-Ramírez, T. | Further evidence of the C-terminal domain chaperon activity in large subunit catalases using unfolded proteins by urea, hydrogen peroxide and heat as models. | Nava-Ramírez, T., Hansberg, W. (2020). Chaperone activity of large-size subunit catalases. Free Radic Biol Med. 20;156:99-106. doi: 10.1016/j.freeradbiomed.2020.05.020. |
| Ortega, I. | Bioinformatic and functional analysis demonstrating the elements found in the signal transduction pathway in *S. schenckii* involved in peroxide sensing. | Ortega, I., Soares Felipe, M.S., Vasconcelos, A.T., Lopes Bezerra, L.M., Da Silva Dantas, A. (2015). Peroxide sensing and signaling in the *Sporothrix schenckii* complex: an in silico analysis to uncover putative mechanisms regulating the Hog1 and AP-1 like signaling pathways. Med Mycol. 53(1):51-9. doi: 10.1093/mmy/myu069. |
| Passardi, F. | This report shows the intricated evolutionary pattern of catalase-peroxidase genes in bacteria and fungi. The complex transfer suggests that these genes are evolutionary complex. | Passardi, F., Zamocky, M., Favet, J., Jakopitsch, C., Penel, C., Obinger, C., Dunand, C. (2007). Phylogenetic distribution of catalase-peroxidases: are there patches of order in chaos? Gene. 1;397(1-2):101-13. doi: 10.1016/j.gene.2007.04.016. |
| Pradhan, A. | This study discusses the role of catalase and the iron acquisition as a double edge sword between surviving ROS and iron scavenging mechanisms. | Pradhan, A., Herrero-de-Dios, C., Belmonte, R., Budge, S., Lopez Garcia, A., Kolmogorova, A., Lee, K.K., Martin, B.D., Ribeiro, A., Bebes, A., Yuecel, R., Gow, N.A.R., Munro, C.A., MacCallum, D.M., Quinn, J., Brown, A.J.P. (2017). Elevated catalase expression in a fungal pathogen is a double-edged sword of iron. PLoS Pathog. 22;13(5):e1006405. doi: 10.1371/journal.ppat.1006405. |
| Román-Casiano, K.M. | One of the few studies addressing the expression and enzymatic activity of catalases in *S. schenckii*. Here, the differential expression and activity are shown. | Román-Casiano, K.M., Martínez-Rocha, A.L., Romo-Lozano, Y., López-Rodríguez, A., Cervantes-García, D., Sierra-Campos, E., Cuéllar-Cruz, M., Ruiz-Baca, E. (2021). Enzyme activity and expression of catalases in response to oxidative stress in *Sporothrix schenckii*. Microb Pathog. 161(Pt B):105270. doi: 10.1016/j.micpath.2021.105270. |
| Saucedo-Campa, D.O. | Proteomic analysis showing novel moonlight proteins such as the Hsp70-5, lipase 1 (Lip1), enolase (Eno), and pyruvate kinase (Pk) as part of the induced proteins in *S. schenckii* exposed to hydrogen peroxide. | Saucedo-Campa, D.O., Martínez-Rocha, A.L., Ríos-Castro, E., Alba-Fierro, C.A., Escobedo-Bretado, M.A., Cuéllar-Cruz, M., Ruiz-Baca, E. (2022). Proteomic Analysis of *Sporothrix schenckii* Exposed to Oxidative Stress Induced by Hydrogen Peroxide. Pathogens. 10;11(2):230. doi: 10.3390/pathogens11020230. |
| Sutay Kocabas, D. | Authors report the identification of a catalase with phenol oxidase activity in a thermophilic fungus. | Sutay Kocabas, D., Bakir, U., Phillips, S.E.V., McPherson, M.J., Ogel, Z.B. (2008) Purification, characterization, and identification of a novel bifunctional catalase-phenol oxidase from *Scytalidium thermophilum*. Applied Microbiology and Biotechnology. 79:407-415. DOI: 10.1007/ s00253-008-1437-y |
| Valenzuela-Cota, D.F. | Effect of an antifungal fraction obtained from the plant *Jacquinia macrocarpa*, showing inhibition of catalases in the fungus *Fusarium verticillioides*. | Valenzuela-Cota, D.F., Buitimea-Cantúa, G.V., Plascencia-Jatomea, M., Cinco-Moroyoqui, F.J., Martínez-Higuera, A.A., Rosas-Burgos, E.C. (2019). Inhibition of the antioxidant activity of catalase and superoxide dismutase from *Fusarium verticillioides* exposed to a *Jacquinia macrocarpa* antifungal fraction. J Environ Sci Health B. 54(8):647-654. doi: 10.1080/03601234.2019.1622978. |
| Ziccardi, M. | Evidence of differential catalase activity in clinical isolates in *Candida parapsilosis*. | Ziccardi, M., Souza, L.O., Gandra, R.M., Galdino, A.C., Baptista, A.R., Nunes, A.P., Ribeiro, M.A., Branquinha, M.H., Santos, A.L. (2015). *Candida parapsilosis* (sensu lato) isolated from hospitals located in the Southeast of Brazil: Species distribution, antifungal susceptibility and virulence attributes. Int J Med Microbiol. 305(8):848-59. doi: 10.1016/j.ijmm.2015.08.003. |
