# Supplementary figures and images for: Catalases in the pathogenesis of Sporothrix schenckii research
Source: PeerJ. 2022 Dec 7;10:e14478. doi: 10.7717/peerj.14478 (PMC9745942; doi:10.7717/peerj.14478)

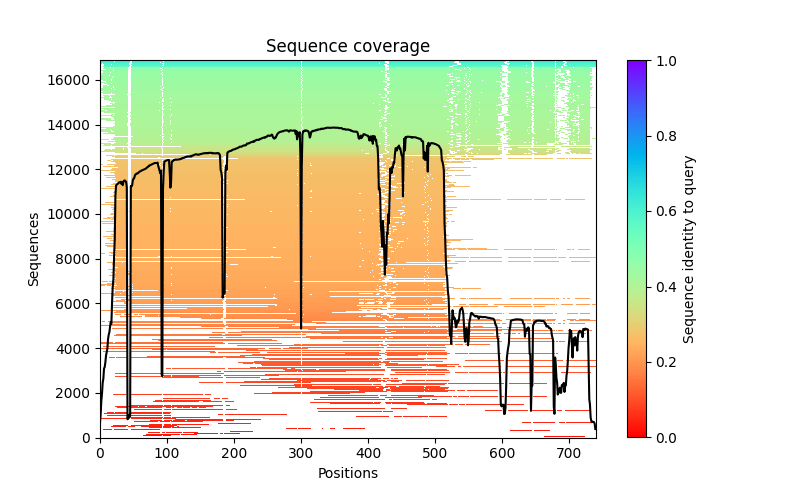

Supplement: Supplemental Information 3 — Sequences and PDB files used in this study. [file peerj-10-14478-s003.zip › Raw data/Cat1 SS/test_106f0_coverage.png]

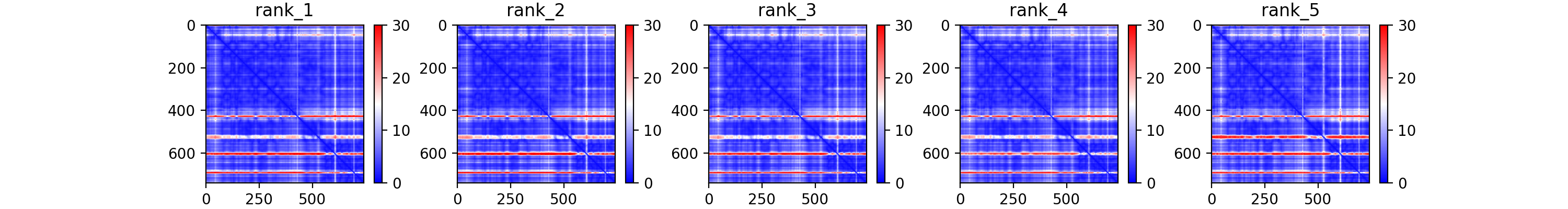

Supplement: Supplemental Information 3 — Sequences and PDB files used in this study. [file peerj-10-14478-s003.zip › Raw data/Cat1 SS/test_106f0_PAE.png]

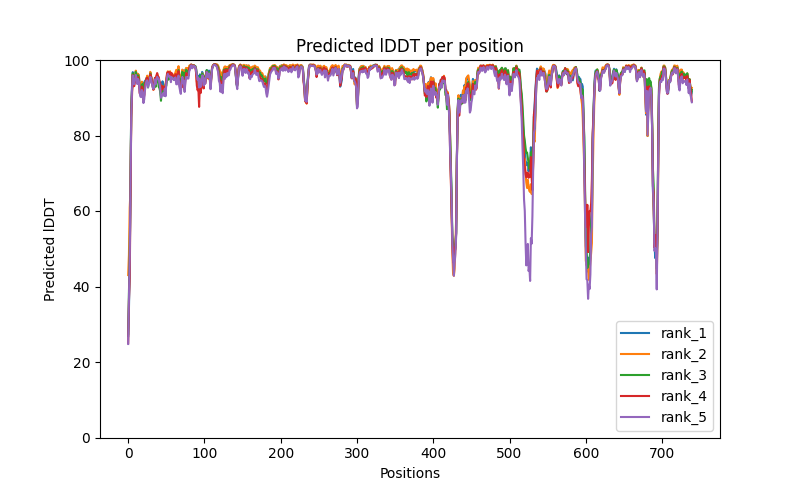

Supplement: Supplemental Information 3 — Sequences and PDB files used in this study. [file peerj-10-14478-s003.zip › Raw data/Cat1 SS/test_106f0_plddt.png]

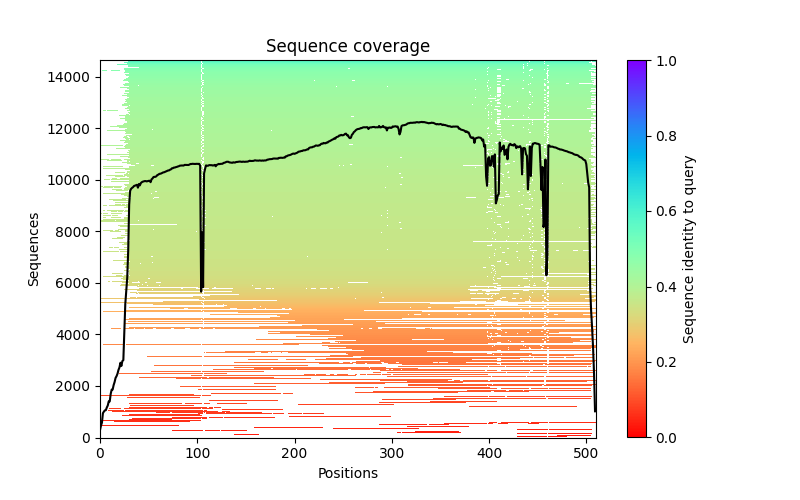

Supplement: Supplemental Information 3 — Sequences and PDB files used in this study. [file peerj-10-14478-s003.zip › Raw data/Cat2 Ss/test_10250_coverage.png]

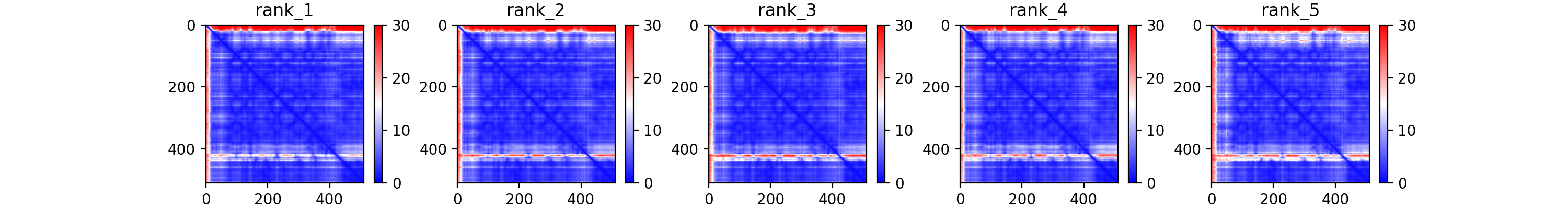

Supplement: Supplemental Information 3 — Sequences and PDB files used in this study. [file peerj-10-14478-s003.zip › Raw data/Cat2 Ss/test_10250_PAE.png]

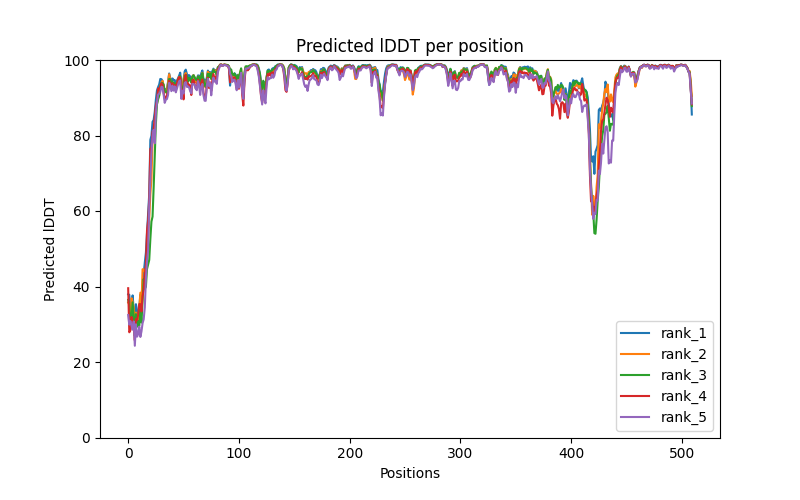

Supplement: Supplemental Information 3 — Sequences and PDB files used in this study. [file peerj-10-14478-s003.zip › Raw data/Cat2 Ss/test_10250_plddt.png]

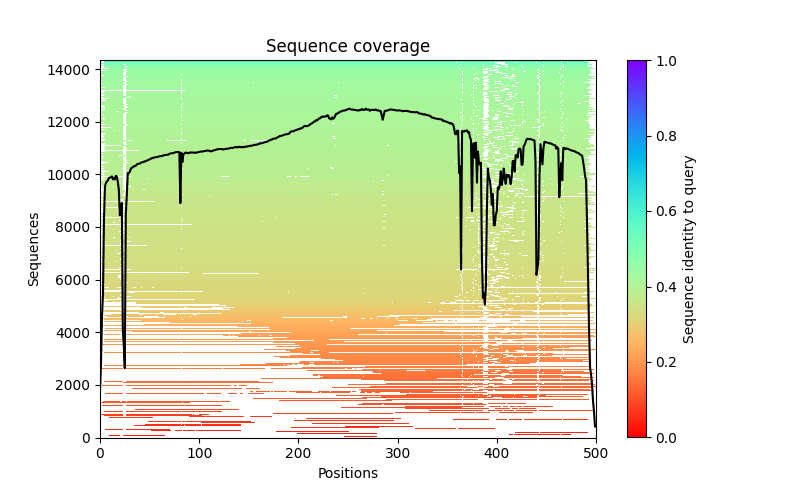

Supplement: Supplemental Information 3 — Sequences and PDB files used in this study. [file peerj-10-14478-s003.zip › Raw data/Cat3 ss/test_991eb_coverage.png]

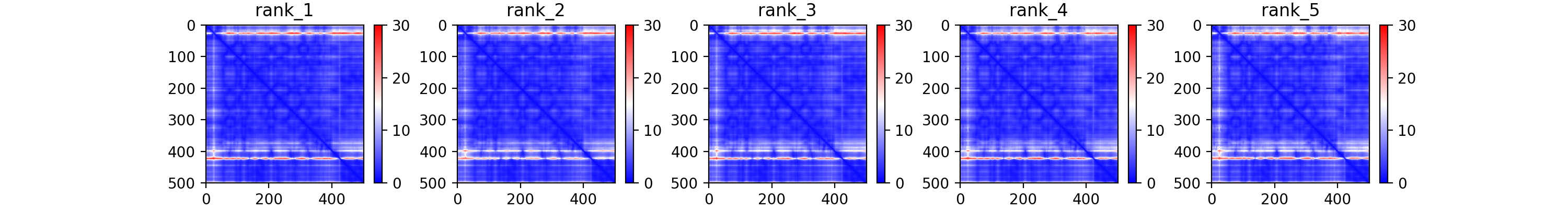

Supplement: Supplemental Information 3 — Sequences and PDB files used in this study. [file peerj-10-14478-s003.zip › Raw data/Cat3 ss/test_991eb_PAE.png]

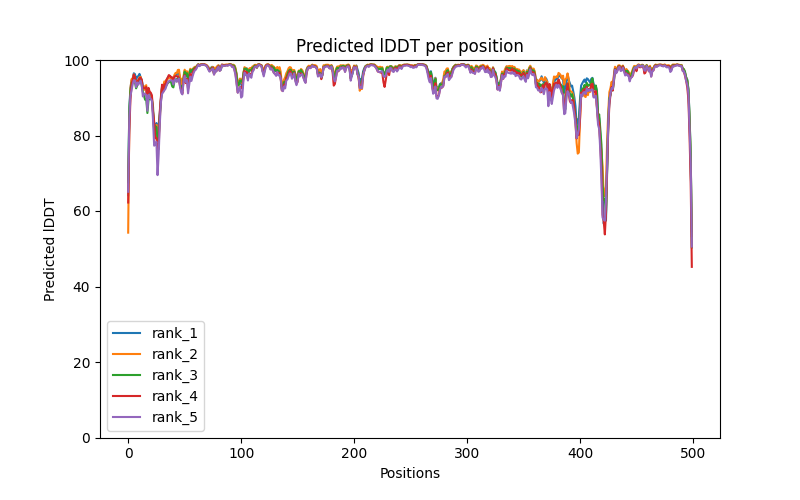

Supplement: Supplemental Information 3 — Sequences and PDB files used in this study. [file peerj-10-14478-s003.zip › Raw data/Cat3 ss/test_991eb_plddt.png]
